# Determining the Function of Social Referencing: The Role of Familiarity and Situational Threat

**DOI:** 10.3389/fpsyg.2020.538228

**Published:** 2020-12-15

**Authors:** Samantha Ehli, Julia Wolf, Albert Newen, Silvia Schneider, Babett Voigt

**Affiliations:** ^1^Mental Health Research and Treatment Center (MHRTC), Faculty of Psychology, Ruhr-University Bochum, Bochum, Germany; ^2^Department of Philosophy II, Ruhr-University Bochum, Bochum, Germany

**Keywords:** social referencing, social-cognitive, information seeking, comfort seeking, co-regulation, infants, familiarity, situational threat, understanding others

## Abstract

In ambiguous situations, infants have the tendency to gather information from a social interaction partner to regulate their behavior [social referencing (SR)]. There are two main competing theories concerning SR’s function. According to social-cognitive information-seeking accounts, infants look at social interaction partners to gain information about the ambiguous situation. According to co-regulation accounts, infants look at social interaction partners to receive emotional support. This review provides an overview of the central developments in SR literature in the past years. We focus on the role of situational aspects such as familiarity of SR partners and situational threat, not only for SR (looking), but also for subsequent behavioral regulation (exploration, affect). As the competing accounts make different predictions concerning both contextual factors, this approach may reveal novel insights into the function of SR. Findings showed that a higher familiarity of SR partners consistently resulted in decreased looking (cf. social-cognitive accounts) and that higher threat remains largely understudied, but seemed to increase looking in the first few studies (cf. co-regulation accounts). Concerning behavioral regulation (exploration, affect) findings are mixed. We point out that moving toward a more complex situatedness may help to disentangle the heterogeneous results by considering the interaction between familiarity and threat rather than investigating the factors in isolation. From a general perspective, this review underlines the importance of situational factors and their interaction in eliciting a phenomenon, such as SR, but also in determining the nature of the phenomenon itself.

## Introduction

Social referencing (SR) is the tendency of a subject (infant) to gather information from an informant (social interaction partner) in order to regulate one’s behavior towards an ambiguous referent for which a fully accurate evaluation is missing (Zarbatany and Lamb, 1985; Walden and Kim, 2005; Striano et al., 2006; Stenberg, 2009; Fawcett and Liszkowski, 2015; Schieler et al., 2018). It emerges from the age of 7 to 10 months and forms a foundation for social learning and social appraisal in adulthood (Walle et al., 2017).

Despite a long tradition of SR research rooting back to the 1980s, there is an ongoing debate concerning the function of SR in infancy. In the classical social-cognitive view, infants refer to other persons in order to seek for information. This perspective is still the default to some extent today, but there are empirical challenges to this view. In 1996, Baldwin and Moses provided a seminal review of SR research in infancy. According to them, the empirical evidence for the classical social-cognitive view could also be fully explained by less demanding processes such as comfort seeking (co-regulation accounts). They recommended taking a situated perspective, that is, examining how the features of the referent and the features of the informant influence SR. Specifically, going beyond an individualistic cognitive approach, they called for research on two questions: How does the (1) familiarity of the SR partner and (2) situational threat influence SR?^1^ As the accounts make different predictions about the influence of these two contextual conditions, the answer to these questions could provide critical novel insights into the function of SR, Baldwin and Moses (1996) argued.

**Figure 1:**
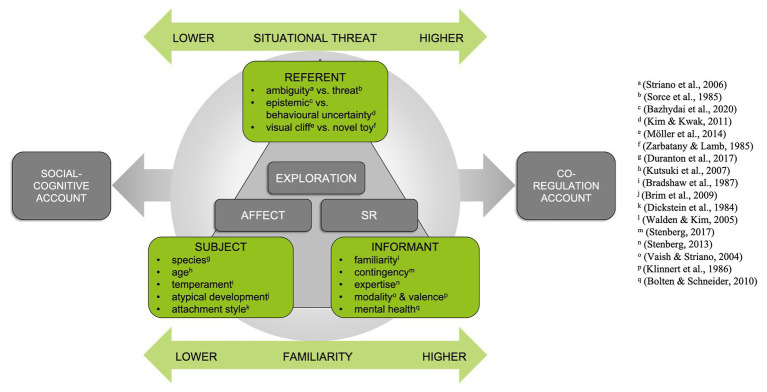
The interplay among features of subject, informant and referent during social referencing (SR). The subject refers to the informant to gather information about the referent (SR). The informant’s reactions influence subject’s affect and the exploration of the referent. Several features of the subject, informant, and the referent have already been examined or are under suspicion to influence SR. The present mini-review focuses on the role of situational threat and familiarity to find out under which circumstances infants refer to the informant in order to gather information (social-cognitive account) or in order to receive emotional support (co-regulation account).

In the past 24 years, several follow-up studies examined how the features of the informant and the referent affect SR. Figure 1 briefly summarizes respective research. However, pursuing Baldwin and Moses (1996) idea, we specifically review research about the role of familiarity of the SR partner and situational threat and evaluate its implications for understanding SR’s function. Mastering the ambiguous referent thereby means that children approach the ambiguous situation (exploration behavior) and/or that children express less negative affectivity (after referring to the informant). Thus, for conclusions about SR’s function, the consideration of exploration behavior and affectivity is of critical importance (Carver and Vaccaro, 2007).

Before drawing conclusions regarding SR’s function, we first describe the two SR-accounts and their predictions for the role of both contextual factors for SR and for infants’ subsequent behavioral regulation (exploration of the referent and infants’ affective expressions). Based on the example of these two contextual features, we will show that an increased sensitivity for the situatedness of SR is a key development in the field of SR. Finally, we discuss how a situated perspective may help disentangling whether a child’s reason to refer to a SR-partner depend on the social and physical context.

## Theoretical Accounts and Their Predictions for the Influence of Familiarity and Situational Threat

### Social-Cognitive Accounts

According to social-cognitive accounts, SR refers to children’s search for information from the SR partner in order to evaluate an ambiguous situation (also referred to as classic information-seeking or information-gathering accounts; Bandura, 1992). These accounts imply that even very young children understand others as sources of information; that is, infants actively seek information before it is provided or even cause others to share their knowledge. Baldwin and Moses (1996) questioned this assumption given infant’s poor performance in explicit theory of mind (ToM) tasks (Wellman et al., 2001). However, more recent findings suggest that infants pass implicit ToM tasks (see Scott, 2017 for a review). Further, evidence on pointing indicates that very young children understand ostensive gestures and use them to interrogate knowledgeable, but not ignorant, social partners (e.g., Liszkowski et al., 2008; Kovács et al., 2014). Thus, infants seem to possess the prerequisites for seeking information. This weakens Baldwin and Moses’ hesitations towards social-cognitive accounts, which remains the most prominent explanation for SR in the current literature (e.g., Shaffer and Kipp, 2014; Meins, 2017).

Only recently, representatives of this theory considered social situational aspects such as familiarity of the SR partner. They predict that infants increase their looking toward more unfamiliar SR partners, as (a) they have a general preference for novel stimuli (novelty hypothesis, Roder et al., 2000), (b) they need more time to understand reactions of more unfamiliar SR partners (familiarity hypothesis, Stenberg, 2012), or (c) the experimenter is usually unfamiliar, but also more knowledgeable with regard to the laboratory context (expertise hypothesis, Feinman et al., 1992).

Such looking preference should lead to behavioral regulation (exploration) in accordance with the message of more unfamiliar SR partners, because the reactions of the preferred SR partner are more salient to the infant. However, consequences of familiarity for infants’ affective expression are largely neglected by social-cognitive accounts and related studies (e.g., Striano and Rochat, 2000; Stenberg and Hagekull, 2007).

According to Baldwin and Moses (1996), making a context more threatening decreases its ambiguity, so that less information is needed to disambiguate the situation. Thus, social-cognitive accounts propose that SR and exploration should decrease with increasing threat. In the case of familiarity, they do not explicitly address how threat would affect infants’ affectivity.

### Co-regulation Accounts

Co-regulation accounts assume that children refer to social partners in order to seek comfort, to check for proximity, or to share affective experiences. These behaviors are not specific to ambiguous situations, but may also occur under these specific circumstances. For example, infants may refer to their mother as ambiguous situations usually elicit arousal, and infants have only limited skills to downregulate this arousal on their own (Kopp, 1989). Thus, SR is seen as one strategy for emotional regulation, in addition to seeking for physical proximity. From this perspective, SR bases on attachment processes (e.g., Ainsworth, 1992) and requires less advanced cognitive skills (Baldwin and Moses, 1996).

Familiarity plays a prominent role in co-regulation accounts. Familiar SR partners, particularly the mother, are seen as a secure base, which helps to maintain infants’ arousal within an optimal range. Familiar interaction partners are usually more competent providers of emotional comfort as infants already learned to trust them and as familiar faces are easier to process (Stenberg and Hagekull, 2007; Ainsworth et al., 2015). In contrast to social-cognitive accounts, co-regulation accounts predict increased looking behavior to more familiar interaction partners and behavioral regulation in accordance with their reaction. This effect is not limited to primary caregivers but, if given the choice, children will generally prefer to look to more familiar SR partners. Further, threatening situations should increase children’s arousal and their need for emotion regulation, resulting in increased SR, increased negative affect, and less exploration.

In short, both accounts consider situational factors, but make different predictions concerning the role of familiarity and threat (Table 1). In the next section, we review relevant findings to evaluate these predictions and their implications for the nature of SR.

**Table 1:** Predictions for the influence of familiarity of the social interaction partner and situational threat on SR, exploration behavior and affectivity according to the social-cognitive accounts, and the co-regulation accounts.

	Social-cognitive accounts	Co-regulation accounts
**Familiarity**		
SR	unfamiliar > familiar	unfamiliar < familiar
Exploration behavior	in line with reactions of unfamiliar informant	in line with reactions of familiar informant
Negative affectivity		
**Potential threat (lower ambiguous threat vs. higher ambiguous threat)**		
SR	lower > higher	lower < higher
Exploration behavior	lower > higher	lower > higher
Negative affectivity	lower = higher	lower < higher

Concerning familiarity, social-cognitive accounts propose that infants should increase looking towards a more unfamiliar person (novelty hypothesis/expertise hypothesis). As the behavior of this person becomes more salient for the infant, infants’ behavioral regulation (exploration) should align with the reaction of the more unfamiliar person (e.g., more exploration, if the unfamiliar person provides a positive message). Co-regulation accounts propose that infants increase looking toward more familiar SR partners, resulting in behavioral regulation in accordance with more familiar SR partners’ reactions to the referent (e.g., more exploration and less negative affect in case of a positive message). Concerning situational threat, social-cognitive accounts propose that SR and exploration should decrease with increasing threat (hence decreasing ambiguity), as less information is needed to disambiguate the situation. In contrast, co-regulation accounts propose increasing SR and negative affectivity and decreasing exploration with increasing threat, as there is a higher need for emotion regulation.

## Empirical Findings for the Influence of Familiarity on SR

The majority of research focuses on looking behavior as the core element of SR. Theoretical accounts of SR imply that children’s search for information aims at dissolving the ambiguous situation. In empirical investigations, the ambiguous situation either refers to a novel toy (e.g., Mumme et al., 1996) or to a visual cliff (e.g., Striano et al., 2006). Extending the work of Baldwin and Moses (1996; who focused only on looking behavior), this review also takes into account SR’s consequences for exploration and negative affectivity. All presented empirical evidence is based on data from children younger than 24 months.

### Looking Behavior

Children generally increase their looking behavior towards other persons in an ambiguous situation to gather information (Carver and Vaccaro, 2007). As predicted by social-cognitive accounts, the majority of studies found that infants preferred to look at an unfamiliar experimenter compared to their looking behavior toward the mother (Walden and Kim, 2005; Stenberg and Hagekull, 2007; Stenberg, 2009; Kim and Kwak, 2011; Schieler et al., 2018). In only one exception that infants looked longer toward the familiar experimenter compared to an unfamiliar experimenter (Stenberg, 2012). Overall, these findings seem to support social-cognitive accounts. However, several concerns remain unresolved.

First, SR is only one of several strategies that infants use to overcome an ambiguous situation; seeking proximity is another. With unfamiliar SR partners the strategy of choice might be increased social looking, whereas with familiar partners it could be proximity seeking where children’s looking pattern remains unaffected. Supporting this idea, Dickstein et al. (1984) found that social looking toward the mother decreases when proximity toward the mother increases, Ainsworth (1992) anecdotally described similar behavior in the strange situation task. Thus, physical proximity to the mother in the studies cited above may have biased the results toward social-cognitive accounts.

Second, it remains open whether familiarity or expertise explains the pattern in favor for social-cognitive accounts, as both features were conflated in most previous studies. Usually, the more experienced experimenter had more interaction time with the child or more speaking time. Evidence directly addressing expertise as an underlying factor for children’s looking preference is mixed. In favor for the expertise account, Stenberg (2012, 2013) found that children preferred to look at the SR partner with more expertise if familiarity was kept constant. Another study attempted to examine familiarity and expertise further by testing some children in the laboratory and some at home (Schieler et al., 2018). In the laboratory, the experimenter might be considered the expert, while at home, the parent should have more expertise. Against the expertise hypothesis, Schieler et al. (2018) found increased looking toward the more unfamiliar experimenter in both contexts, even at home. Nonetheless, children might still have seen the experimenter as the expert, who instructed the parent (Walden and Kim, 2005). Hence, the question of familiarity vs. expertise as critical factor remains to be clarified by future studies.

Third, more fine-grained analyses of looking pattern data revealed that despite the preference for more unfamiliar interaction partners, infants increased looking behavior toward both the experimenter and the mother, when the former presented a novel toy (Schmitow and Stenberg, 2013). Infants seem to need reassurance from more familiar interaction partners to trust the information provided by unfamiliar, yet more knowledgeable SR partners. One interpretation may be that co-regulative and social-cognitive functions complement each other, a possibility that has not been tested empirically so far.

### Exploration Behavior

While evidence of looking behavior seems to support social-cognitive accounts, the few findings relating to children’s exploration behavior (of the ambiguous situation) are mixed. Stenberg and Hagekull (2007) found that children explored more with the unfamiliar experimenter than with the mother. Extending this evidence to other levels of familiarity, Schmitow and Stenberg (2013) found more exploration of a novel toy when it was presented by the unfamiliar experimenter as opposed to the familiar experimenter.

Analogous to looking behavior, expertise could explain the effect of familiarity in these studies (but see Zmyj et al., 2012, for contradictory findings in the context of imitation). Indeed, Stenberg (2013) showed that children increased their exploratory behavior more after receiving information from the expert experimenter. Here too the proximity to the mother (as a secure base and source for emotional comfort) may have biased the exploratory pattern in the direction predicted by social-cognitive accounts. However, both points cannot explain the results of studies that found the opposite pattern supporting co-regulative accounts. In those studies, children only approached the ambiguous situation after receiving information from their parent (Schieler et al., 2018) or a more familiar experimenter compared to a less familiar experimenter (Stenberg, 2012). Other studies even found contradictory findings, depending on which kind of exploratory behavior was analyzed. In Stenberg (2009), children looked more at a novel toy if the information was provided by the mother, but played more with it when the information came from the unfamiliar experimenter.

Taken together, it seems that infants show less (e.g., Schmitow and Stenberg, 2013), more (Schieler et al., 2018), or different explorative behavior (Stenberg, 2009) in the presence of their mother compared to an unfamiliar SR partner. However, when exploring familiarity independent of expertise, expertise seems to have the critical impact on the exploration behavior (Stenberg, 2012). Further, infants’ behavior seems to be more affected by negative reactions of the social partner compared to positive ones (Vaish et al., 2008; Schieler et al., 2018), which may have obscured the influence of familiarity in some studies.

Thus, the current pattern for exploration behavior does not clearly speak in favor of one account. It must be borne in mind that the effect of SR on exploratory behavior is measured in much fewer studies, while measuring looking behavior is required for any SR paradigm. Hence, the heterogeneous findings result from a weak empirical base and await clarification in future studies.

### Affect

In the context of SR research, affectivity could reflect an adequate emotional reaction to the ambiguous situation after receiving information about it (social-cognitive accounts). Alternatively, maybe emotional displays reflect the result of emotion regulation (co-regulation accounts).

The co-regulative pattern of lower negative affectivity in the presence of more familiar interaction partners receives little empirical support. Most studies found no significant differences in affect in the presence of SR partners of different familiarity (Walden and Kim, 2005; Carver and Vaccaro, 2007; Stenberg, 2009, 2012; Kim and Kwak, 2011). Usually, children showed relatively low levels of distress in any condition within the respective studies. Such low variability may explain the absence of effects on affectivity.

Overall, the findings about the influence of familiarity draw an inconclusive picture varying between and within domains (SR, exploration, and affectivity). Hence, whether SR’s function aligns with the predictions of the social-cognitive or co-regulation account still remains open. Baldwin and Moses (1996) suggested a crucial role of situational threat as a second contextual factor, which could resolve the contradictory findings above.

## Empirical Evidence for the Influence of Situational Threat on SR

Even though Baldwin and Moses already proposed in 1996 that situational threat might provide new insights on SR’s function, only little progress has been made in this regard. In the few available studies, infants showed higher SR, less exploration (crossed a visual cliff less often, Striano et al., 2006), and increased negative affect (higher levels of arousal, Schwartz et al., 1973) on a steeper cliff (i.e., more threatening, less ambiguous) in comparison to a flatter cliff (i.e., less threatening, more ambiguous). Striano and Rochat (2000) found the same effect in a novel toy paradigm where they used a toy dog and measured infants’ SR before the dog barked (lower potential threat) and after the dog barked (higher potential threat). SR increased with increasing threat. This supports co-regulation accounts that assume children should generally increase SR as one method of comfort seeking in highly threatening contexts. Besides the potential threat of a referent, other possible features have been neglected in research. For example, it might be interesting to assess the differences resulted by visual cliff vs. novel toy paradigms, as the former seems to have direct implications for infants’ behavior (Figure 1). Findings from both ambiguous tasks have been used interchangeably.

## Conclusion and Outlook

Baldwin and Moses have been pioneers in suggesting a stronger situatedness in investigating SR. This has led to a new direction in SR research, and respective findings give rise to new questions. In this review, we summarized research about the influence of two situational factors on SR – namely familiarity and threat. Baldwin and Moses proposed that the examination of both contextual factors (independent of each other) could help to elucidate SR’s function. We reviewed respective research of the past 24 years leading to three major findings. First, higher familiarity of an interaction partner consistently resulted in decreased looking in many studies (in line with social-cognitive accounts). Second, only few studies examined the impact of familiarity on infants’ subsequent exploration and affectivity with contradictory results. Third, situational threat remains largely neglected in empirical research, but seemed to influence SR, exploration, and affectivity in the few available studies (in line with co-regulation accounts). Thus, the function of SR may be more complex than previously suggested.

To resolve this puzzle, we suggest extending Baldwin and Moses’ ideas and moving on from a simple situatedness to a more complex situatedness. This means not only considering both contextual factors independently, but also addressing the impact of familiarity in situations of different levels of threat. Rethinking the predictions of both accounts from this perspective results in new hypotheses. Social-cognitive accounts predict less relevance for SR as information-seeking strategy if the situation becomes more threatening. Hence, the preference to look at less familiar social partners should become less evident with increasing threat. In turn, co-regulation accounts assume more relevance of SR (as emotion regulation strategy) if the situation becomes more threatening. Thus, the looking preference for more familiar interaction partners should become particularly apparent with increasing situational threat. In other words, SR may serve different functions, depending on the current context conditions: shifting from information-seeking in highly ambiguous, less threatening conditions to emotion regulation in ambiguous but more clearly threatening contexts (Figure 1). Thus, we suggest that the question is not whether SR serves a social-cognitive or co-regulative function, but rather under what circumstances which function prevails.

Research examining the interplay of both contextual factors is missing so far, but we propose that this is a key strategy for clarifying the inconclusive findings about the function of SR. On a conceptual level, respective evidence would (a) unify both so far competing accounts on a higher hierarchical level and (b) underline the importance of a situated perspective for understanding the complex context-dependent nature of well-known developmental phenomena such as SR. Research investigating additional contextual factors that might modulate the role of SR would be a second promising avenue.

## Author Contributions

SE, JW, AN, SSch, and BV contributed conception and structure of this mini-review. SE and JW compiled the draft of the manuscript. BV wrote sections of the manuscript. All authors contributed to manuscript revision, read and approved the submitted version.

### Conflict of Interest

The authors declare that the research was conducted in the absence of any commercial or financial relationships that could be construed as a potential conflict of interest. One editor of this special issue is also co-author of this publication; this did not compromise the impartiality as we submitted a blinded manuscript and suggested a non-affiliated editor for this publication. JW and AN as well as SE, AN, SSch, and BV have collaborated on a publication within the past 2 years. AN is a PhD-supervisor of JW and SE; SS is supervising SE.

## Footnotes

^1^Authors raise a third question concerning which modalities (facial, bodily, verbal) influence SR to elaborate on the intentionality behind SR. As the question of intentionality is not in the focus of the present review, we do not address literature on the influence of modalities here.
